# Kindlin-2 maintains liver homeostasis by regulating GSTP1–OPN-mediated oxidative stress and inflammation in mice

**DOI:** 10.1016/j.jbc.2023.105601

**Published:** 2023-12-28

**Authors:** Yiming Zhong, Liang Zhou, Hui Wang, Sixiong Lin, Tiemin Liu, Xingxing Kong, Guozhi Xiao, Huanqing Gao

**Affiliations:** 1 Shanghai Key Laboratory of Metabolic Remodeling and Health, State Key Laboratory of Genetic Engineering, Institute of Metabolism and Integrative Biology, School of Life Sciences, Jinshan Hospital, Fudan University, Shanghai, China; 2 Department of Biochemistry, School of Medicine, Guangdong Provincial Key Laboratory of Cell Microenvironment and Disease Research, Shenzhen Key Laboratory of Cell Microenvironment, Southern University of Science and Technology, Shenzhen, China; 3 School of Life Sciences, Sun Yat-sen University, Guangzhou, China; 4 Guangdong Provincial Key Laboratory of Orthopedics and Traumatology, Department of Spinal Surgery, The First Affiliated Hospital of Sun Yat-sen University, Guangzhou, China

**Keywords:** Kindlin-2, hepatocyte, oxidative stress, osteopontin, GSTP1

## Abstract

Hepatocyte plays a principal role in preserving integrity of the liver homeostasis. Our recent study demonstrated that Kindlin-2, a focal adhesion protein that activates integrins and regulates cell–extracellular matrix interactions, plays an important role in regulation of liver homeostasis by inhibiting inflammation pathway; however, the molecular mechanism of how Kindlin-2 KO activates inflammation is unknown. Here, we show that Kindlin-2 loss largely downregulates the antioxidant glutathione-*S*-transferase P1 in hepatocytes by promoting its ubiquitination and degradation *via* a mechanism involving protein–protein interaction. This causes overproduction of intracellular reactive oxygen species and excessive oxidative stress in hepatocytes. Kindlin-2 loss upregulates osteopontin in hepatocytes partially because of upregulation of reactive oxygen species and consequently stimulates overproduction of inflammatory cytokines and infiltration in liver. The molecular and histological deteriorations caused by Kindlin-2 deficiency are markedly reversed by systemic administration of an antioxidant *N*-acetylcysteine in mice. Taken together, Kindlin-2 plays a pivotal role in preserving integrity of liver function.

Liver is comprised of parenchymal cells and nonparenchymal cells, which play critical roles in body homeostasis. Liver diseases are a worldwide health care problem. Hepatic fibrosis is a reversible healing response characterized by the accumulation of extracellular matrix (ECM) following liver injury. However, a sustained injury causes chronic inflammation and ECM accumulation and leads to a progressive substitution of liver parenchyma by scar tissue. This process results in cirrhosis, the end consequence of progressive fibrosis, which usually have a poor outcome and high mortality. Hepatocyte death and inflammation are two central components during the development of liver fibrosis. Mice with Tnfr1 or Trail deficiency are protected against cholestatic liver injury, supporting a mechanistic role for death receptor family members in obstructive cholestasis (1). Recent studies reported that dying hepatocytes generate signals that initiate a sterile inflammatory response in cells of the innate immune system, leading to further damage on neighboring hepatocytes and initiating a crosstalk with hepatic stellate cells (HSCs) (2, 3). Furthermore, activation and differentiation of HSCs upregulate expression of transforming growth factor β1 and collagen 1 through Toll-like receptor 9 in HSCs and promotes liver fibrosis (4). Reactive oxygen species (ROS) play an important role to program liver fibrosis development. Previous studies indicate that ROS derived from cytochrome p450 2E1 (*Cyp2e1*) of hepatocytes can induce alpha 2 collagen type I (*Col1α2*) gene expression in HSCs (5, 6). In addition, ROS derived from Kupffer cells contribute to liver fibrosis by activating HSCs (7). In addition, ROS stimulate apoptosis of hepatocytes and exacerbate the inflammatory response by activating proinflammatory pathways (8).

Osteopontin (OPN), an ECM protein, is highly expressed in inflamed tissues and plays a critical role in wound healing (9). Previous studies demonstrate that OPN is significantly upregulated in HSCs during liver fibrosis (10, 11). Furthermore, transgenic mice expressing OPN in hepatocytes developed spontaneous fibrosis over time, whereas ablation of OPN alleviated liver fibrosis (12). OPN is an oxidative stress–sensitive cytokine, which could drive fibrogenesis *via* integrin and PI3K–pAkt–NF-κB signaling pathway (12). Nonetheless, mechanisms through which hepatocyte injury causes inflammation and fibrosis remain poorly understood.

Kindlin proteins consist of three members, Kindlin-1, Kindlin-2, and Kindlin-3. Kindlin proteins play key roles in the regulation of cell–ECM adhesion, differentiation, and migration (13, 14, 15). Kindlin-2 is implicated in a number of biological and pathological processes, such as cell differentiation, survival, organogenesis, and carcinogenesis (16, 17, 18, 19, 20, 21, 22, 23, 24, 25). Our previous study showed that Kindlin-2 plays an important role in maintaining liver function by inhibiting inflammation activation (26). Deletion of Kindlin-2 resulted in liver injury and premature death. However, the molecular mechanism of how Kindlin-2 KO activates inflammation is unknown.

Here, by utilizing a combination of multiomics profiling and biochemical and genetical approaches, we found that loss of Kindlin-2 in hepatocyte downregulated glutathione-*S*-transferase P1 (GSTP1), the liver-detoxifying enzyme, resulted in immune cell infiltration and ROS production, and aggravated liver damage. These results suggest Kindlin-2–GSTP1 axis as a potential therapeutic target for liver injury and associated diseases.

## Results

### Multiomics profiling reveals hepatocyte mitochondrial damage and enhanced liver dysfunction in Kindlin-2 hepatocyte KO mice

**Figure 1:**
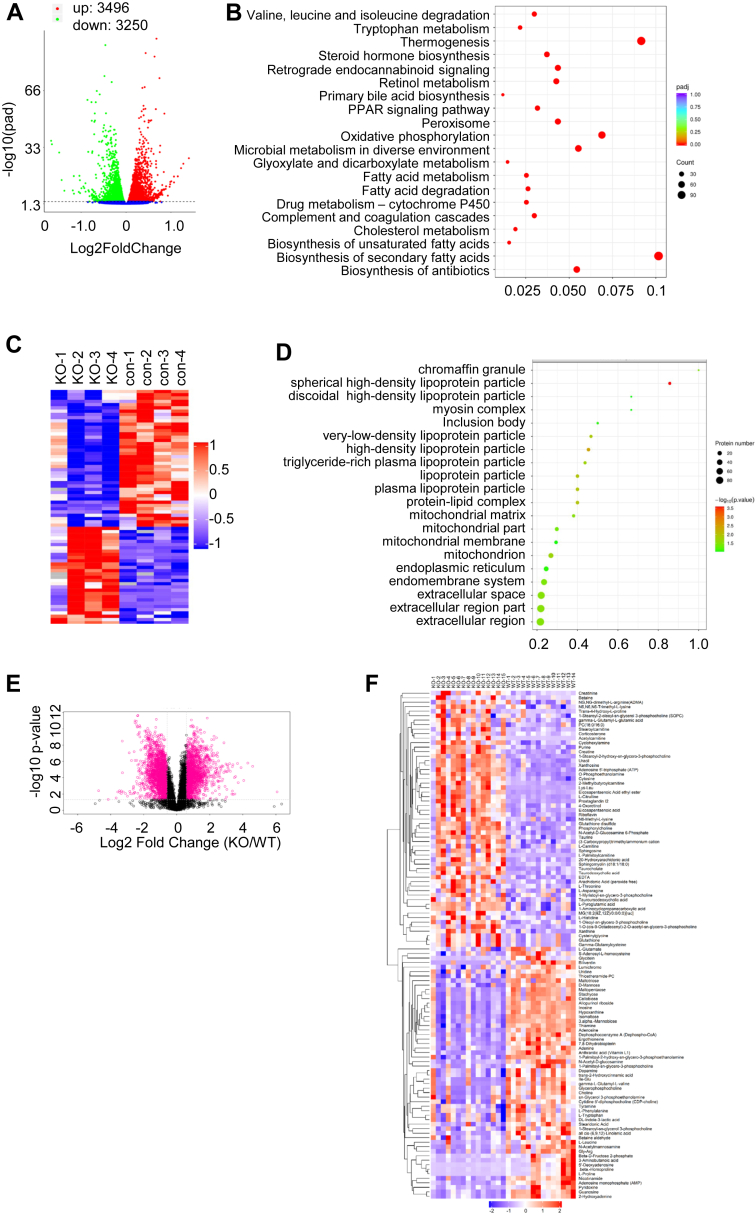
**Multiomics profiling reveals hepatocyte mitochondrial damage in Kindlin-2 hepatocyte KO mice.***A* and *B*, transcriptomic alteration. *A*, volcano map of DEGs. *B*, KEGG enrichment analysis of the biological processes. *C* and *D*, serum proteomic alteration. *C*, volcano plot showing upregulated (*red*) and downregulated (*blue*) proteins in KO *versus* control sera. *D*, GO cellular component analysis. *E* and *F*, liver metabolomic alteration. *E*, volcano diagram of differential metabolites. *F*, heatmap of the significantly different metabolites. DEG, differently expressed gene; GO, Gene Ontology; KEGG, Kyoto Encyclopedia of Genes and Genomes.

Our recent study showed that deletion of Kindlin-2 in hepatocyte caused liver dysfunction and premature death through activating inflammation signal (26). To further determine the impact of Kindlin-2 loss on hepatocyte function at the molecular level, we performed RNA-Seq analyses from 4-week-old control and KO liver and identified 3496 upregulated genes and 3250 downregulated genes (Fig. 1*A*). Kyoto Encyclopedia of Genes and Genomes analysis revealed that Kindlin-2 loss impacted multiple pathways involved in cholesterol and fatty acid metabolism, thermogenesis, and oxidative phosphorylation (Fig. 1*B*). Gene Ontology enrichment analysis revealed that mitochondrial NADH dehydrogenase activity, transmembrane transport, ATP production, electron transportation, and oxidative phosphorylation were largely impaired in KO *versus* control livers (Table S1). In the meantime, glycolytic process was enhanced in KO *versus* control livers (Table S2).

We next performed proteomics analyses of sera from 4-week-old control and KO mice. We identified 67 proteins that exhibited statistically significant changes and differential expression, including 30 upregulated proteins and 37 downregulated proteins in KO *versus* control sera (Tables S3 and S4). Among upregulated proteins, cytochrome *c*, which is released from mitochondria and involved in apoptosis, was increased in KO sera compared with that in control sera. Furthermore, the levels of multiple apolipoproteins (A-I, A-II, C-I, C-II, C-III, C-IV, and M), which are primarily synthesized by hepatocytes and participate in lipids and cholesterol transportation and metabolism, were downregulated in KO *versus* control sera. In addition, we identified 57 proteins that were only detected in KO sera and 16 proteins only in control sera (Table S5 and S6). For example, carbamoyl phosphate synthase-1 and glutamate dehydrogenase 1, both serum markers for mitochondrial damage, were detected only in KO sera. Similarly, interferon alpha/beta receptor 2, which is involved in inflammation, was only identified in KO but not control sera. In contrast, prohibitin, which is critical for integrity of the mitochondrial structure, was not detected in KO sera. Cellular component analysis revealed that Gene Ontology terms associated with “mitochondrial” were largely affected (Fig. 1, *C* and *D*).

We finally performed metabolic profiling of 4-week-old control and KO liver tissues and found that a total of 29 metabolites were significantly downregulated (Fig. 1, *E* and *F* and Table S7), and 21 metabolites were significantly upregulated in KO *versus* control mice (Table S8). Downregulated metabolites in KO livers included amino acids and derivatives (proline, leucine, and γ-aminobutanoic acid), sugars (maltopentaose, *N*-acetylmannosamine, and *N*-acetyl-d-glucosamine), lipids (linolenic acid and glycerophosphocholine), nucleosides (inosine, hypoxanthine, and adenosine), vitamins or coenzyme (pyridoxine and 8-dihydrobiopterin), and biliverdin (Table S7). Several important metabolites were significantly upregulated in KO *versus* control liver tissues, including acetylcarnitine, a marker for mitochondrial damage, and prostaglandin I2, which promotes inflammation. l-citrulline, a key intermediate of the urea cycle, was largely upregulated in KO relative to that in control livers, suggesting altered ammonia assimilation. l-palmitoylcamitine was accumulated in KO *versus* control livers, which may be related to reduced fatty acid biosynthesis by KO liver. The level of ATP was elevated in KO *versus* control liver tissue extracts, which is probably because of reduced ATP consumption caused by impaired anabolic metabolism (Table S8).

### Kindlin-2 loss impairs liver function partially by promoting ROS production and oxidative stress

**Figure 2:**
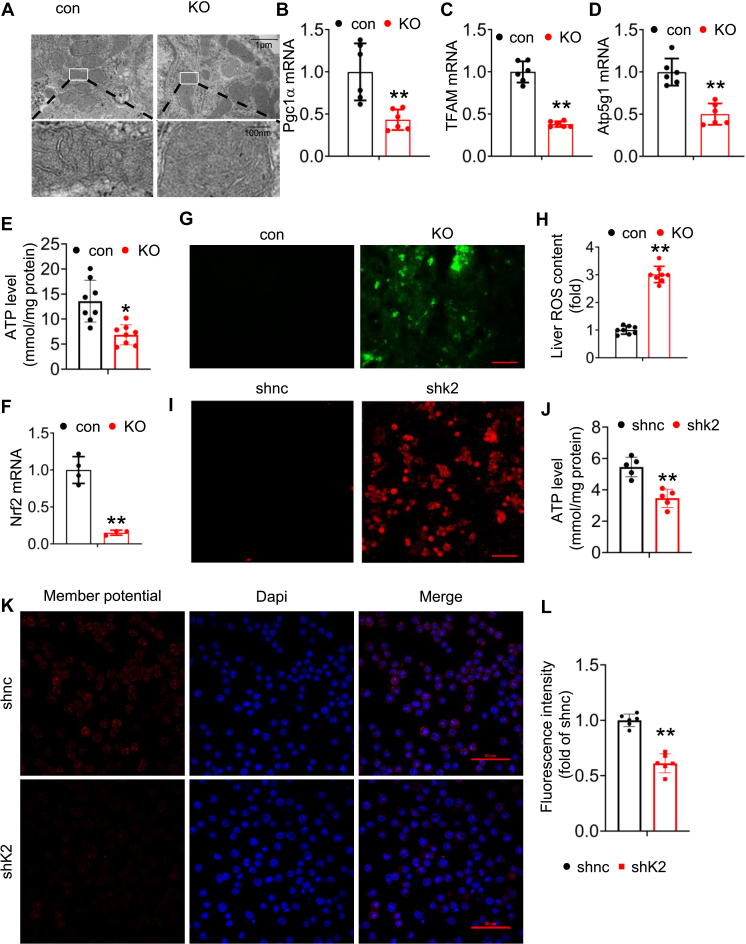
**Kindlin-2 loss promotes mitochondrial damage and oxidative stress.***A*, transmission electron micrograph (TEM) showing hepatocyte mitochondrial damage in 4-week-old KO mice. Scale bar represents 1 μm. *B*, quantitative real-time RT–PCR (qRT–PCR) analysis for expression of Pgc1α in liver tissues from 4-week-old control and KO mice (N = 6 mice/group). *C*, qRT–PCR analysis for expression of TFAM in liver tissues from 4-week-old control and KO mice (N = 6 mice/group). *D*, qRT–PCR analysis for expression of Atp5g1 in liver tissues from 4-week-old control and KO mice (N = 6 mice/group). *E*, ATP detection of 4-week-old control and KO mice (N = 8 mice/group). *F*, qRT–PCR analysis for Keap1 and Nrf2 expression in livers from 4-week-old control and KO mice (N = 4 mice/group). *G*, dichlorofluorescin diacetate fluorescence assay showing increased ROS production in KO livers. Scale bar represents 100 μm. *H*, fresh liver tissue ROS was measured by using a ROS assay kit in control and KO mice (N = 8 mice/group). *I*, dichlorofluorescin diacetate fluorescence assay showing increased ROS production in adeno-Cre-infected *Kinldlin-2*^*fl/fl*^ hepatocytes. Scale bar represents 100 μm. *J*, ATP detection of shnc and shk2 hepatocytes. *K*–*L*, mitochondrial membrane potential detection. *K*, mitochondrial membrane potential staining in hepatocytes. Scale bar represents 50 μm. *L*, quantification of (*K*). ∗*p* < 0.05, ∗∗*p* < 0.01, *versus* control. Pgc1α, peroxisome proliferator–activated receptor-C coactivator-1A; ROS, reactive oxygen species; TFAM, transcription factor A mitochondrial.

Results from aforementioned multiomics analyses strongly suggest that Kindlin-2 loss severely damages hepatocyte mitochondrial function. To further support this notion, we performed transmission electron micrograph analysis of 4-week-old control and KO liver tissues and observed revealed apparent swollen mitochondria and loss of cristae in KO hepatocytes (Fig. 2*A*). The expression levels of TFAM (transcription factor A mitochondrial), which encodes a key mitochondrial transcription factor A, and peroxisome proliferator–activated receptor-C coactivator-1A, which is critical for mitochondrial biogenesis, were both dramatically decreased in KO livers relative to those in control livers (Fig. 2, *B* and *C*). The expression of Atp5g1, which is one of ATP synthases, is decreased in KO mice liver (Fig. 2*D*). Moreover, ATP level is decreased in KO mice liver (Fig. 2*E*).

Mitochondria are the main source of ROS, which increases oxidative stress. Interestingly, we found that the expression levels of antioxidative genes, including nuclear factor E2–related factor 2 (Nrf2), were decreased in KO livers compared with those in control livers (Fig. 2*F*). Results from both dichlorodihydrofluorescein diacetate staining and ROS concentration measurements showed a dramatic increase in ROS production in KO relative to control liver tissues (Fig. 2, *G* and *H*). Similarly, deleting Kindlin-2 expression increased ROS production and decreased ATP level in hepatocytes *in vitro* (Fig. 2, *I* and *J*). Furthermore, mitochondrial membrane potential staining showed that knockdown of Kindlin-2 in hepatocyte significantly reduced mitochondrial function (Fig. 2, *K* and *L*). Collectively, aforementioned results demonstrate that Kindlin-2 loss promotes mitochondrial damage and increases oxidative stress in hepatocytes.

### Systemic *N*-acetylcysteine administration reverses partial phenotype and extends life span of KO mice

**Figure 3:**
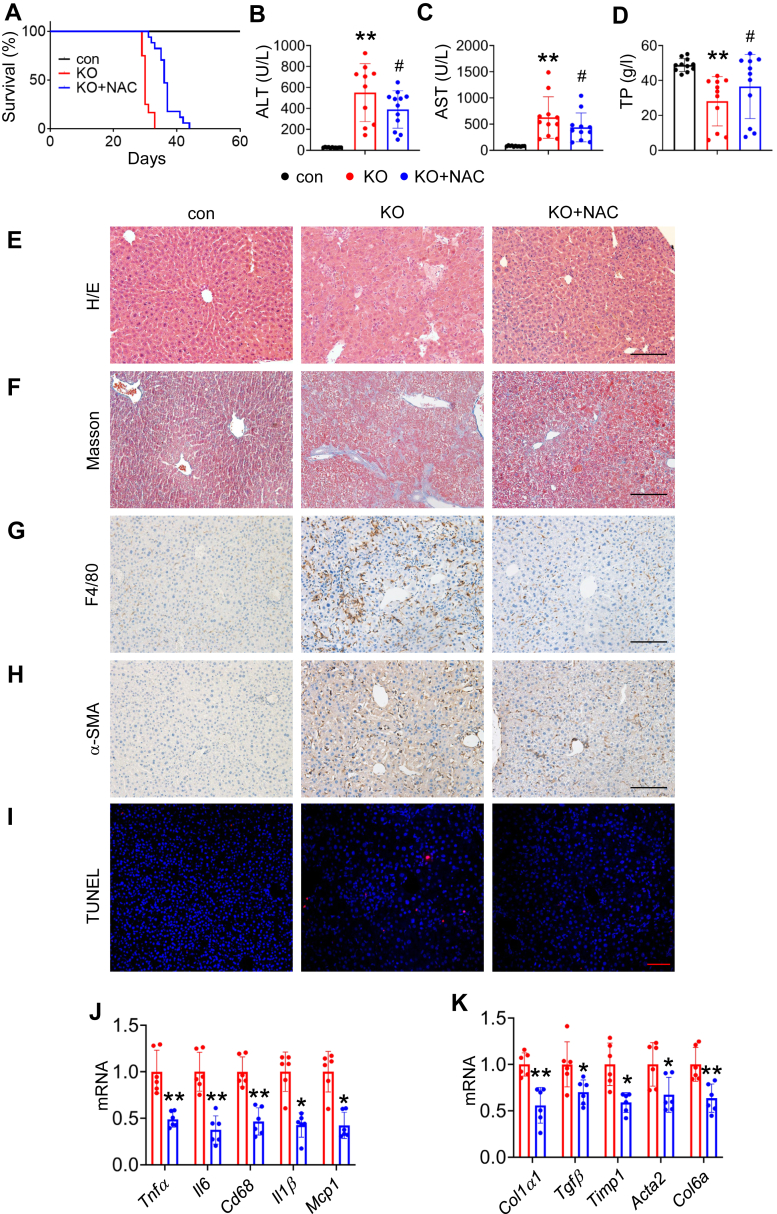
**Systemic NAC administration reverses partial phenotype and extends life span of KO mice.***A*, survival curve. KO mice were supplemented with NAC in the drinking water (10 g/l), starting at postnatal day 18. *B*–*D*, serum ALT, AST, and total protein (TP) levels from KO mice with and without NAC administration at 4 weeks of age (N = 9–12 mice/group). *E*, H&E staining of liver sections of control mice, KO mice with and without NAC administration at 4 weeks of age. Scale bar represents 100 μm. *F*, Masson’s trichrome staining of liver sections of control mice, KO mice with and without NAC administration. Scale bar represents 100 μm. *G*, macrophage infiltration evaluated by F4/80 IHC staining. Scale bar represents 100 μm. *H*, expression of α-SMA was determined by IHC. Scale bar represents 100 μm. *I*, TUNEL staining. Scale bar represents 100 μm. *J* and *K*, qRT–PCR analysis. RNAs isolated from liver tissues of KO and NAC-treated mice were subjected to qRT–PCR analyses for the indicated genes (N = 6 mice/group). ∗*p* < 0.05, ∗∗*p* < 0.01, *versus* KO. ALT, alanine aminotransferase; AST, aspartate aminotransferase; IHC, immunohistochemical; NAC, *N*-acetylcysteine; qRT–PCR, quantitative RT–PCR; α-SMA, alpha-smooth muscle actin.

We next determined whether inhibition of oxidative stress can alleviate the progression of liver dysfunction of KO mice. We found that systemic administration of the anti-ROS *N*-acetylcysteine (NAC) *via* drinking water significantly extended the life span of KO mice (Fig. 3*A*). NAC largely ameliorated hepatocyte function in KO mice, as demonstrated by significant reductions of serum alanine aminotransferase and aspartate aminotransferase levels in KO mice treated with NAC compared with those in KO without NAC treatment (Fig. 3, *B* and *C*). In further support of improved liver function, NAC-treated KO mice displayed higher level of total serum protein than that in untreated KO mice (Fig. 3*D*). At the histological level, NAC attenuated liver structural deterioration in KO mice (Fig. 3*E*) and reduced liver fibrosis (Fig. 3*F*). The macrophage infiltration in KO liver tissues was greatly mitigated by NAC treatment (Fig. 3*G*). NAC treatment largely reduced expression of alpha-smooth muscle actin in KO livers (Fig. 3*H*). Finally, NAC treatment ameliorated apoptosis, which was induced by Kindlin-2 deletion (Fig. 3*I*). Gene detection showed that NAC significantly ameliorated liver inflammation (Fig. 3*J*) and liver fibrosis (Fig. 3*K*). Thus, blocking ROS production reverses liver inflammation, fibrosis, apoptosis, and improves survival of KO mice.

### Kindlin-2 interacts with GSTP1 and inhibits GSTP1 ubiquitination

**Figure 4:**
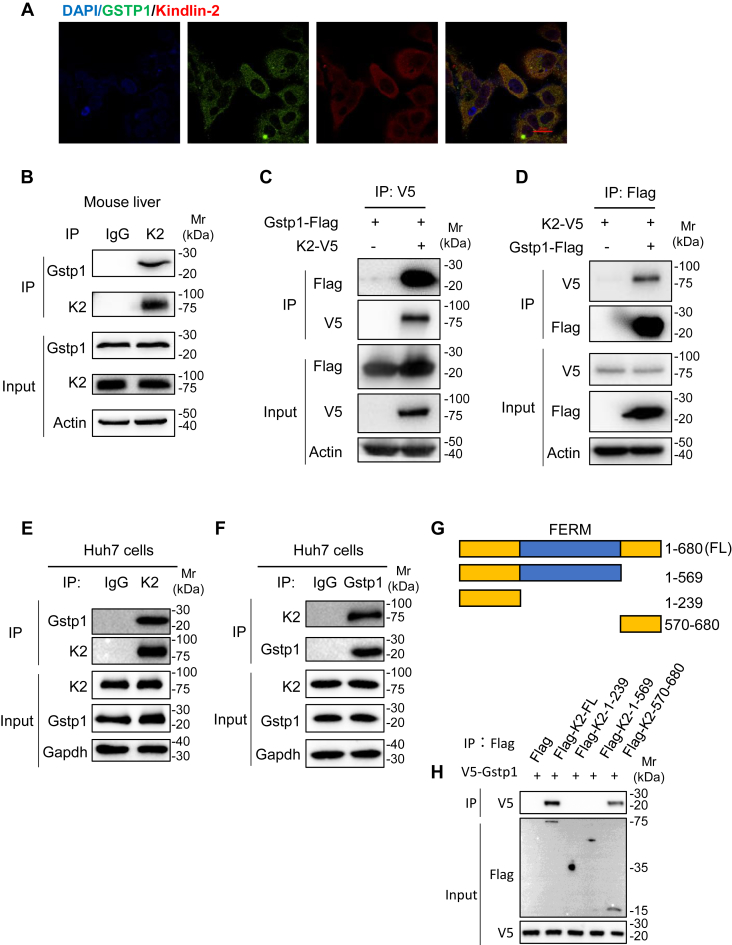
**Kindlin-2 interacts with GSTP1.***A*, IF staining. Huh7 cells were subjected to double immunostaining with anti-GSTP1antibody (*green*) and anti-Kindlin-2 antibody (*red*), followed by visualization with confocal microscopy. Scale bar represents 20 μm. *B*, interaction of Kindlin-2 with endogenous GSTP1 in liver tissue. Liver lysates were used for immunoprecipitation (IP) and immunoblotting (IB) with the antibodies as indicated. *C* and *D*, co-IP assays. Cell lysates from HEK293T cells transfected with FLAG-tagged GSTP1 and/or V5-tagged Kindlin-2 were used for IP and IB with the antibodies as indicated. *E* and *F*, co-IP assays. Huh7 cells were used for IP and IB with the indicated antibodies for the interaction of endogenous Kindlin-2 and GSTP1. *G*, a schematic diagram of the full-length (FL) and truncated Kindlin-2 plasmid constructs. *H*, co-IP assays. HEK293T cells were cotransfected with plasmid constructs expressing GSTP1 and full-length or truncated Kindlin-2. Forty-eight hours later, whole-cell extracts were prepared and subjected to co-IP assays. Co-IP, coimmunoprecipitation; GSTP1, glutathione-*S*-transferase P1; HEK293T, human embryonic kidney 293T cell line; IF, immunofluorescence.

To investigate molecular mechanistically how Kindlin-2 regulates liver metabolism, we performed proteomic analyses as initial screen to identify novel proteins potentially associated with Kindlin-2. Combined with RNA-Seq, serum proteomics, and metabolomics analysis, we targeted GSTP1, which participates in cellular response to oxidative stress, associates with Kindlin-2 to regulate liver homeostasis (27, 28). To confirm this, we stained GSTP1 and Kindlin-2 in Huh-7 cells, and the result showed that Kindlin-2 and GSTP1 colocalized in the cytoplasm (Fig. 4*A*). Next, we used pull-down assay to test this association. As expected, GSTP1 was pulled down by Kindlin-2 (Fig. 4*B*). To further test the association, we performed coimmunoprecipitation (co-IP) experiments with either anti-Kindlin-2 or anti-GSTP1 antibodies. The data showed that GSTP1 was co-IPed with Kindlin-2 (Fig. 4*C*). Reciprocally, Kindlin-2 was co-IPed with GSTP1 (Fig. 4*D*). Moreover, endogenous Kindlin-2 protein was coimmunoprecipitated by an anti-Kindlin-2 antibody and anti-GSTP1 antibody in Huh7 cells (Fig. 4, *E* and *F*). We next generated Kindlin-2 deletion plasmid constructs as indicated (Fig. 4*G*) to define regions within the Kindlin-2 molecule that are essential for its interaction with GSTP1 protein. The results showed that C-terminal region (amino acids 570–680) of Kindlin-2 is necessary for its interaction with GSTP1 (Fig. 4, *G* and *H*).

**Figure 5:**
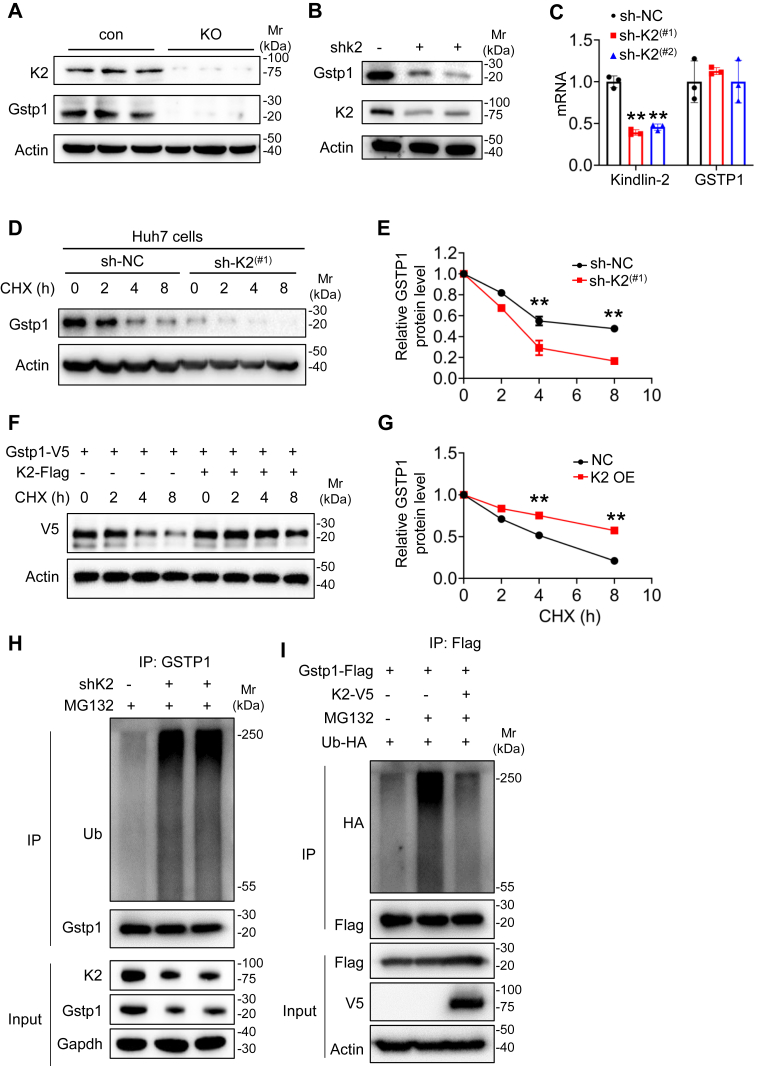
**Kindlin-2 regulates GSTP1 expression by affecting its polyubiquitination.***A*, immunoblotting analysis of GSTP1 protein levels in primary hepatocyte isolated from Kindlin-2 KO and control mice. *B* and *C*, lentivirus-containing control shRNAs or Kindlin-2-specific shRNAs were used to infect Huh7 cells, followed by immunoblotting and PCR to determine GSTP1 protein and mRNA levels. *D*–*G*, cycloheximide (CHX) experiments. Huh7 cells (*D*, *E*) or HEK293T cells (*F*, *G*) with Kindlin-2 shRNA KD or Kindlin-2 plasmid were treated with 100 μg/ml of CHX for the indicated times, followed by Western blotting for expression of GSTP1. *H*, Kindlin-2 knockdown increases endogenous GSTP1 polyubiquitination. Huh7 cells were stably transfected with lentivirus-expressing control shRNAs or Kindlin-2-specific shRNAs. The cells were pretreated with MG132 for 6 h, and then the cell lysates were used in IP and IB with the antibodies as indicated. *I*, Kindlin-2 protects GSTP1 ubiquitination. HEK293T cells were transiently transfected with V5-tagged Kindlin-2, and FLAG-tagged GSTP1 as indicated. At 24 h after the transfection, the cells were pretreated with or without MG132 for 6 h, followed by IP and IB with antibodies as indicated. GSTP1, glutathione-*S*-transferase P1; HEK293T, human embryonic kidney 293T cell line; IB, immunoblotting; IP, immunoprecipitation; KD, knockdown.

Next, we examined GSTP1 protein expression in Kindlin-2 KO hepatocyte and Kindlin-2 knockdown hepatocyte. Immunoblotting showed that the level of GSTP1 protein was reduced in both KO and knockdown hepatocyte compared with control (Fig. 5, *A* and *B*), and the GSTP1 mRNA level was unchanged (Fig. 5*C*). We next performed the cycloheximide experiments and found that Kindlin-2 knockdown dramatically decreased the GSTP1 protein stability in Huh7 cells (Fig. 5, *D* and *E*), and Kindlin-2 overexpression increased the GSTP1 protein stability in human embryonic kidney 293T (HEK293T) cells (Fig. 5, *F* and *G*). To determine whether Kindlin-2 could affect the degradation of GSTP1 protein, we analyzed GSTP1 protein polyubiquitination levels in the presence or the absence of MG132, a proteasome inhibitor. In the presence of MG132, knockdown of Kindlin-2 significantly increased GSTP1 polyubiquitination levels in Huh7 cell (Fig. 5*H*), and GSTP1 polyubiquitination levels were decreased by Kindlin-2 overexpression in HEK293T cells (Fig. 5*I*), indicating that Kindlin-2 could decrease GSTP1 ubiquitination and protect its degradation.

### Kindlin-2 loss stimulates hepatic macrophage activation and inflammation by upregulating OPN

**Figure 6:**
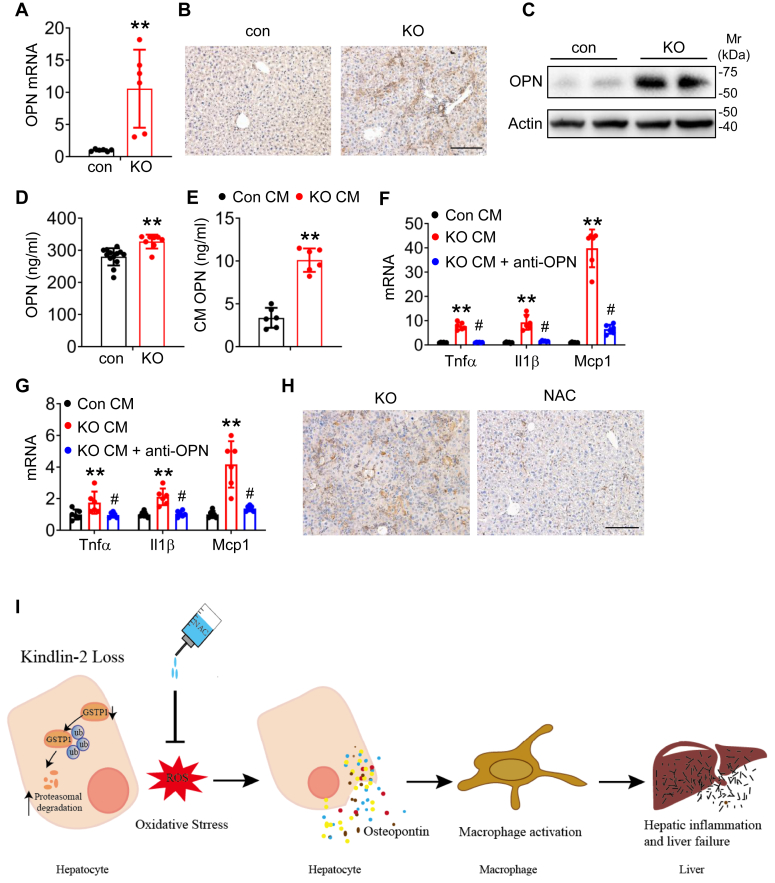
**Kindlin-2 loss stimulates hepatic macrophage activation and inflammation by upregulation of osteopontin.***A*, quantitative real-time RT–PCR (qPCR) analyses. RNAs isolated from liver tissues of 4-week-old control and KO mice were subjected to qPCR analyses (N = 6 mice/group). *B*, immunohistochemical (IHC) staining. Four-week-old control and KO mouse liver sections were subjected to IHC staining using an anti-OPN antibody. Scale bar represents 100 μm. *C*, Western blotting. Protein extracts from 4-week-old control and KO mouse liver tissues were subjected to Western blotting for expression of OPN. *D*, serum OPN level was measured in 4-week-old control and KO mice using an ELISA kit (N = 10 mice/group). *E*, conditioned media (CM) from control and KO primary hepatocytes were subjected to ELISA for OPN. *F* and *G,* qRT–PCR analyses. The CM from control and KO hepatocytes were added onto cultured primary Kupffer cells (*F*) or RAW 264.7 cells (*G*) in the absence or the presence of 10 μg/ml of OPN-neutralizing antibody (anti-OPN) for 48 h. The mRNA levels of *Tnfα*, *Il1β*, and *Mcp1* were determined by qRT–PCR analyses. *H*, IHC staining for OPN of KO mice with and without NAC administration. Scale bar represents 100 μm. *I*, a schematic illustrating how Kindlin-2 regulates liver homeostasis. Figure created using BioRender.com. ∗*p* < 0.05, ∗∗*p* < 0.01, *versus* control; ^##^*p* <. 0.01, *versus* KO. NAC, *N*-acetylcysteine; OPN, osteopontin.

Previous studies demonstrate that OPN, a key oxidative stress–sensitive cytokine, is upregulated in fibrosis livers (12). Interestingly, we found that the mRNA and protein levels of OPN were dramatically increased in KO liver compared with those in control liver, as determined by quantitative PCR analysis, Western blotting, and immunohistochemical staining (Fig. 6, *A*–*C*). Consistent with increased expression of OPN in liver, serum level of OPN was significantly elevated in KO mice relative to that in control mice (Fig. 6*D*). Furthermore, the level of OPN significantly increased in conditioned media (CM) from KO primary hepatocytes contained significantly higher level of OPN than that from control cells (Fig. 6*E*).

Because OPN is a proinflammatory cytokine and plays an important role in regulating tissue remodeling (29), we wondered whether increased OPN production from KO hepatocyte is responsible for hepatic macrophage activation and inflammatory response induced by Kindlin-2 loss. We next determined whether OPN plays a role in hepatic macrophage activation. We collected the CM from control and KO hepatocytes. Then, Kupffer cells or RAW 264.7 cells were treated with the CM in the absence or the presence of 10 μg/ml of OPN-neutralizing antibody for 48 h. We observed that CM from KO hepatocyte increased the expression of proinflammatory cytokines in both Kupffer cells (Fig. 6*F*) and RAW 264.7 cells (Fig. 6*G*). Importantly, addition of the OPN-neutralizing antibody to the cultures essentially abolished the ability of KO CM to stimulate expression of the proinflammatory cytokines in both Kupffer cells and RAW 264.7 cells (Fig. 6, *F* and *G*). Also, blocking of ROS by NAC significantly decreased OPN expression (Fig. 6*H*). Taken together, our findings suggest a paracrine mechanism in which hepatocyte Kindlin-2 deletion induces the production of OPN, which subsequently activates macrophage proinflammatory response (Fig. 6*I*).

## Discussion

In the present study, we identified how Kindlin-2 loss in hepatocyte activates inflammation pathway, leading to liver damage. Kindlin-2 loss causes GSTP1 degradation, leading to hepatocyte mitochondrial damage and promotes excessive production of ROS and oxidative stress, which stimulates OPN production and macrophage activation, inflammation, and apoptosis.

We provide convincing evidence that Kindlin-2 ablation results in hepatocyte mitochondrial damage and increases ROS production. Hepatocyte mitochondria from KO mice are swollen and display reduced cristae, where electron transport and oxidative phosphorylation take place. Results from our multiomics profiling also suggest impaired mitochondrial function by the loss of Kindlin-2, as demonstrated by reduced expression of mitochondrial NADH dehydrogenase and enzymes involved in transmembrane transport, electron transportation, and oxidative phosphorylation in KO relative to control hepatocytes. The serum markers for mitochondrial damage, including carbamoyl phosphate synthase-1 (30) and glutamate dehydrogenase (31), are upregulated in KO mice, whereas prohibitin, which protects mitochondrial function (32), is not detected in KO sera. Recent evidence suggests that hepatic mitochondrial dysfunction is involved in steatohepatitis (33). Interestingly, we recently reported that a fraction of Kindlin-2 protein is present in mitochondria in a lung cancer cell line (34).

GSTP1 is one of the major members of GST family of metabolic enzymes, which involves in detoxifying carcinogens of cellular defense system (35). Mouse has two GSTP genes, *Gstp1* and *Gstp2* (36), and *Gstp1* is much more abundant than *Gstp2* in mouse liver (37). GSTP^−/−^ mice are more susceptible to stress-induced organ injuries, exhibiting aggravated oxidative stress, inflammation, apoptosis, and higher mortality (38, 39). Previous studies have shown that extracellular GSTP1 can cross plasma membrane, and rGSTP1 protects against lipopolysaccharide-induced acute liver damage and lung injury by reducing apoptosis and inflammation (37, 40). Overexpression of GSTP1 mitigated ROS accumulation and protected oxidative stress–induced cell death, and application of antioxidant NAC partially recovered the activity of GSTP1 (27, 37). Here, we demonstrate that Kindlin-2 loss increases intracellular ROS probably by reducing the level of GSTP1 protein in hepatocytes. We find that Kindlin-2 binds to GSTP1 and inhibits its ubiquitination. Recent study reported that FBX8, which has E3 ligase activity, could degrade GSTP1 (41). How Kindlin-2 binding to GSTP1 inhibits GSTP1 ubiquitination? This should be investigated in the future study.

Our studies suggest that Kindlin-2 loss promotes inflammatory response by upregulating, at least in part, expression of OPN in hepatocytes. This notion is supported by the following lines of evidence from this and other studies. First, studies by other groups have established a critical role of OPN in pathogenesis of liver fibrosis (42). OPN is largely upregulated in patients with liver fibrosis (11, 12, 43). Second, in the present study, we showed that Kindlin-2 loss in hepatocytes induces dramatic expression of fibrogenic genes and causes massive liver fibrosis in mice. Third, our *in vitro* and *in vivo* studies reveal that Kindlin-2 loss dramatically increases OPN expression in hepatocytes. Finally, the upregulation of inflammatory cytokines in Kupffer cells and RAW 264.7 cells induced by CM from Kindlin-2-deficienct hepatocytes is essentially abolished by an anti-OPN-neutralizing antibody. In further support of the aforementioned notion, OPN was reported to promote hepatic inflammation by modulating multiple signaling pathways in hepatocytes, HSCs, and other cell types (44). Syn *et al*. (10) reported that activation of Hh signaling in HSCs increased OPN expression *via* Gli2 transcription factor in mouse model of nonalcoholic steatohepatitis. Interestingly, Raquel *et al*. (12) showed that OPN was responsive to ROS in HSCs.

It is important to point out that mitochondria are the major organelle that produces ROS (45, 46). We find that Kindlin-2 loss dramatically increases ROS production in hepatocytes. Excessive ROS is known to interfere with the normal function of liver cells and plays a critical role in the pathogenesis of liver fibrosis (47). ROS-activated HSCs undergo a phenotypic switch and deposit excessive amount of ECM that alters the normal liver architecture and affects liver function (48). In addition, ROS stimulates necrosis and apoptosis of hepatocytes, which causes liver injury and leads to the progression of liver disease (49). Interestingly, systemic administration of the antioxidant NAC markedly ameliorates histological and metabolic abnormalities in KO mice. It should be pointed out that antioxidants have been a mainstay of therapeutic strategy for chronic liver diseases. Previous studies have showed that mitochondrial function is ameliorated following NAC treatment (50, 51).

Based on our results, we propose that Kindlin-2 interacts with GSTP1 and inhibits GSTP1 ubiquitination. Thus, Kindlin-2 loss decreases the level of GSTP1 protein in hepatocytes, leading to elevation of intracellular ROS and oxidative stress. Excessive oxidative stress stimulates OPN production, which stimulates macrophage activation and inflammation, resulting in hepatocyte apoptosis. Hepatocyte death causes proliferation of nonhepatocytes, which produces and accumulates excessive ECM, eventually leading to liver fibrosis and liver failure. Given that fibrosis is the major pathological characteristic of chronic liver diseases, Kindlin-2 may be a novel therapeutic target for these diseases.

While our studies showed that deletion of Kindlin-2 increased OPN secretion, it is important to determine whether hepatocyte-specific deletion of the *Osteopontin* (*SPP1*) gene expression can rescue the phenotypes in Kindlin-2 KO mice in future study.

## Experimental procedures

### Animal study

Generation of Kindlin-2^fl/fl^ mice has been reported previously (52). In order to eliminate the expression of Kindlin-2 in hepatocytes, Kindlin-2^fl/fl^ mice were hybridized with Alb-Cre transgenic mice to produce Kindlin-2^fl/fl^; Alb-Cre mice, that is, the hepatocyte conditional Kindlin-2 KO mice (hereinafter referred to as KO). This study used Cre-negative floxed Kindlin-2 mice (*i.e.*, Kindlin-2^fl/fl^) as the control. All animal experiments were conducted at the Southern University of Science and Technology's specific pathogen-free experimental animal center. The mice were fed with NAC (Sigma, 10 g/l) in drinking water every 2 days (53). All animal protocols in this study were approved by the Institutional Animal Care and Use Committees of the Southern University of Science and Technology.

### Biochemical measurements

Blood was collected from mice inferior vena cava under anesthesia with isoflurane. After 2 h of solidification at room temperature, serum was collected by centrifugation at 4 °C. Serum aspartate aminotransferase, alanine aminotransferase, and albumin were measured with commercial kits (Shensuoyoufu).

### Histological analyses

Tissues were fixed with 4% paraformaldehyde (PFA) and then embedded in paraffin. About 5 μm paraffin sections were used for subsequent staining. H&E staining, Matson's trichrome staining, and Sirius red staining were performed as previously described (26).

### Immunofluorescence staining

Immunofluorescence staining was performed as previously described (54). Briefly, cells were fixed with 4% PFA for 30 min, permeabilized by 0.3% Triton X-100, and closed for 30 min (catalog no.: P0260; Beyotime). After overnight incubation with anti-Gstp1 or anti-Kindlin-2 antibodies, cells were incubated with Alexa Fluor 488/568-labeled secondary antibodies and 4′,6-diamidino-2-phenylindole (catalog no.: P0265; Beyotime).

### ROS analysis

Liver frozen sections were fixed in 4% PFA. Tissue sections were incubated with either dihydroethidium (5 μM; Thermo Fisher) or dichlorodihydrofluorescein diacetate (5 μM; Abcam) for 30 min at 37 °C in a humidified chamber protected from light. Images were acquired by fluorescence microscopy. Liver tissue ROS was measured using an assay kit (catalog no.: GMS10016; GENMED).

### IP

IP was performed as previously described (55). Specifically, total protein lysate of cells or liver tissues was extracted with lysis buffer. The supernatant fractions were separated by centrifugation at 13,000*g* for 10 min and subjected to IP using the indicated antibodies at 4 °C overnight. The beads were washed three times and then heated at 95 °C in loading buffer for 10 min and subjected to immunoblotting as described later in detail.

### Western blot analysis

Total protein samples were extracted from tissues or cells, and protein concentration was measured using BCA Protein Assay Kit (catalog no.: P0010S; Beyotime). Protein samples were separated by SDS-PAGE and immunoblotted with the indicated primary antibodies and corresponding secondary antibody (56, 57). Blots were developed with chemiluminescent horseradish peroxidase substrate. Antibody information are listed in Table S9. Blots were quantified by using ImageJ software (National Institutes of Health).

### Hepatic metabolomics analysis

Liver metabolites were extracted in 80% methanol. Mass spectrometry (MS) polar metabolite analysis was performed at Shanghai Applied Protein Technology Co Ltd. MS analyses were performed using an UHPLC (1290 Infinity LC; Agilent Technologies) coupled to a quadrupole time-of-flight (TripleTOF 6600; AB Sciex).

### Proteomics

The serum quantitative proteomics and immunoprecipitated samples were conducted at Shanghai Applied Protein Technology Co Ltd. LC–MS/MS analysis was performed on a Q Exactive mass spectrometer (Thermo Scientific) that was coupled to Easy nLC (Proxeon Biosystems; now Thermo Fisher Scientific) for 120 min. The instrument was run with peptide recognition mode enabled. The MS data were analyzed using MaxQuant software, version 1.5.3.17 (Max Planck Institute of Biochemistry in Martinsried) (58).

### Quantitative real-time RT–PCR analysis

RNA extraction, reverse transcription, and quantitative PCR analysis were performed as described previously (59). The mRNA expression levels of the target genes were normalized to GAPDH. Primer sequences are listed in Table S10.

### TUNEL assay

TUNEL staining was performed as described previously. After 4% PFA fixation of frozen sections of liver, TUNEL staining was performed using an In-Situ Cell Death Detection Kit (catalog no.: C1088; Beyotime).

### Transmission electron microscopy

After overnight fixation in 2.5% glutaraldehyde in PBS at 4 °C, 1 mm^3^ cubes were removed from the liver, washed three times in PBS, followed by postfixation with 1% OsO_4_. After dehydration, thin sections were stained with uranyl acetate and lead citrate for observation. Sections were imaged using a JEOL JEM 1011 transmission electron microscope.

### Primary hepatocyte isolation and cell culture

Cells were cultured in a humidified incubator at 37 °C and 5% CO_2_. Primary hepatocytes were isolated from 8- to 12-week-old *Kindlin-2*^*fl/fl*^ mice by liver perfusion with type IV collagenase and grown on collagen-coated plates as described previously (55). After overnight culture, cells were incubated for 6 h at 37 °C with a reduced volume of culture medium containing the Cre-adenovirus and then refed with fresh medium to delete Kindlin-2 expression. About 12 h later, cells were subjected to oxidative stress analysis.

Huh7, RAW 264.7, and HEK293T cells were purchased from American Type Culture Collection cultured in Dulbecco’s modified Eagle’s medium (Gibco) supplemented with 10% fetal bovine serum (Gibco), 50 units/ml penicillin, and 50 mg/ml streptomycin at 37 °C, and 5% CO_2_ in a humidified chamber. All cell lines are monitored for mycoplasma using detection kit (Sigma–Aldrich) according to the manufacturer’s instructions.

### Macrophage culture and treatment

Primary Kupffer cells were isolated from mice as described previously (60). For conditioned medium (CM) treatment, hepatocyte CM was collected. After filtration with sterile filters, it was diluted 1:1 (v/v) using Dulbecco's modified Eagle's medium containing 5% fetal bovine serum and then added to cultured Kupffer cells. Mouse OPN-neutralizing antibody (10 g/ml) was preincubated with hepatocyte CM for 2 h and then added to Kupffer cells and RAW cells for 48 h.

### Quantification of OPN by ELISA

Mouse serum and cultured hepatocyte supernatants were collected, and OPN levels were determined using the Mouse/Rat OPN Quantikine ELISA kit (MOST00; R&D Systems).

### Statistical analysis

The results are shown as means ± SD. The unpaired two-tailed Student's *t* test was used to determine significant differences between two groups. *p* < 0.05 was considered statistically significant.

## Data availability

All data are available from the corresponding author upon reasonable request.

## Supporting information

This article contains supporting information.

## Conflict of interest

The authors declare that they have no conflicts of interest with the contents of this article.
