# Supplementary material for: Kindlin-2 maintains liver homeostasis by regulating GSTP1–OPN-mediated oxidative stress and inflammation in mice
Source: J Biol Chem. 2023 Dec 28;300(2):105601. doi: 10.1016/j.jbc.2023.105601 (PMC10831259; doi:10.1016/j.jbc.2023.105601)
Supplement: Supplementary Tables S1 and S10 [file mmc1.docx]

**Kindlin-2 maintains liver homeostasis by regulating GSTP1-OPN-mediated oxidative stress and inflammation in mice**

**Supplementary Table 1. Go enrichment analysis report (down-regulated vs. con)**

| Enrichment of pathway | P value |
| --- | --- |
| oxidation-reduction process | 6.65E-39 |
| oxidoreductase activity, acting on paired donors, with incorporation or reduction of molecular oxygen | 3.93E-21 |
| heme binding | 1.81E-20 |
| iron ion binding | 4.01E-20 |
| catalytic activity | 2.06E-14 |
| oxidoreductase activity | 6.90E-10 |
| mitochondrion | 8.13E-08 |
| ATP synthesis coupled proton transport | 2.94E-07 |
| transmembrane transport | 1.24E-06 |
| zinc ion binding | 1.70E-06 |
| NADH dehydrogenase (ubiquinone) activity | 4.56E-06 |
| oxidoreductase activity, acting on the CH-CH group of donors | 7.42E-06 |
| cytochrome-c oxidase activity | 1.02E-05 |
| ATPase activity, coupled to transmembrane movement of substances | 1.93E-05 |
| flavin adenine dinucleotide binding | 2.90E-05 |
| transferase activity, transferring acyl groups other than amino-acyl groups | 0.000102 |
| mitochondrial inner membrane | 0.000264 |
| ATPase activity | 0.000289 |
| metal ion binding | 0.000327 |
| proton transmembrane transporter activity | 0.000473 |
| iron-sulfur cluster binding | 0.000473 |
| electron transfer activity | 0.000697 |
| hydrolase activity, hydrolyzing O-glycosyl compounds | 0.00117 |
| lipid binding | 0.001323 |
| transferase activity, transferring hexosyl groups | 0.001539 |
| biosynthetic process | 0.001985 |
| oxidoreductase activity, acting on the CH-OH group of donors, NAD or NADP as acceptor | 0.002617 |
| sulfotransferase activity | 0.0057 |
| lipoprotein metabolic process | 0.005707 |

**Supplementary Table 2. Go enrichment analysis report (up-regulated vs. con)**

| Enrichment of pathway | P value |
| --- | --- |
| GTP binding | 5.08E-07 |
| calcium ion binding | 6.54E-07 |
| GTPase activity | 1.47E-06 |
| actin binding | 4.32E-05 |
| phosphatidylinositol binding | 8.24E-05 |
| signal transduction | 0.000148 |
| protein dephosphorylation | 0.000157 |
| regulation of apoptotic process | 0.000208 |
| protein tyrosine phosphatase activity | 0.000218 |
| cell adhesion | 0.000218 |
| antigen processing and presentation | 0.000331 |
| insulin-like growth factor binding | 0.000852 |
| extracellular matrix | 0.001009 |
| glycolytic process | 0.001281 |
| cytoskeleton | 0.001343 |
| regulation of signal transduction | 0.001343 |
| extracellular matrix structural constituent | 0.001757 |
| immune response | 0.001884 |

**Supplementary Table 3. Serum proteomics proteins expression (up-regulated vs con)**

| O35490 | BHMT1 Betaine--homocysteine S-methyltransferase 1 |
| --- | --- |
| P35505 | FAAA Fumarylacetoacetase |
| P28798 | GRN Progranulin |
| P26043 | RADI Radixin |
| O08749 | DLDH Dihydrolipoyl dehydrogenase |
| P33434 | MMP2 72 kDa type IV collagenase |
| Q07456 | AMBP Protein AMBP |
| Q00519;G3X982;Q3TYQ9 | XDH Xanthine dehydrogenase/oxidase |
| P11680 | PROP Properdin O |
| P70663 | SPRL1 SPARC-like protein 1 |
| Q06890 | CLUS Clusterin |
| P11672 | NGAL Neutrophil gelatinase-associated lipocalin |
| Q91Y97 | ALDOB Fructose-bisphosphate aldolase B |
| P62897 | CYC Cytochrome c, somatic |
| P08607 | C4BPA C4b-binding protein |
| Q9JJH1 | RNAS4 Ribonuclease 4 |
| Q9JM99 | PRG4 Proteoglycan 4 |
| P09581 | CSF1R Macrophage colony-stimulating factor 1 receptor |
| Q9R182 | ANGL3 Angiopoietin-related protein 3 |
| P97430 | SLPI Antileukoproteinase |
| P29699 | FETUA Alpha-2-HS-glycoprotein |
| Q61129 | CFAI Complement factor I |
| P51670 | CCL9 C-C motif chemokine 9 |
| P70274 | SEPP1 Selenoprotein P |
| P11276 | FINC Fibronectin |
| P47876 | IBP1 Insulin-like growth factor-binding protein 1 |
| P06909 | CFAH Complement factor H |
| Q9CPY7 | AMPL Cytosol aminopeptidase |
| O09061 | PSB1 Proteasome subunit beta type-1 |
| Q8CIZ8 | VWF von Willebrand factor |

**Supplementary Table 4. Serum proteomics proteins expression (down-regulated vs con)**

| Q61696;P17879 | HS71A Heat shock 70 kDa protein 1A |
| --- | --- |
| Q91WP0 | MASP2 Mannan-binding lectin serine protease2 |
| P22599 | A1AT2 Alpha-1-antitrypsin 1-2 |
| Q01149 | CO1A2 Collagen alpha-2(I) chain |
| P43025 | TETN Tetranectin |
| Q3UV17 | K22O Keratin, type II cytoskeletal 2 oral |
| P09036 | ISK1 Serine protease inhibitor Kazal-type 1 |
| P62984;P62983 | RL40 Ubiquitin-60S ribosomal protein L40 |
| Q9Z1T2 | TSP4 Thrombospondin-4 |
| P97298;Q95121 | PEDF Pigment epithelium-derived factor |
| P23953;Q64176 | EST1C Carboxylesterase 1C |
| Q8K558 | TRML1 Trem-like transcript 1 protein |
| P07758 | A1AT1 Alpha-1-antitrypsin 1-1 |
| Q00897 | A1AT4 Alpha-1-antitrypsin 1-4 |
| A2ASS6 | TITIN Titin |
| Q03734 | SPA3M Serine protease inhibitor A3M |
| Q00623 | APOA1 Apolipoprotein A-I |
| Q8VCG4 | CO8G Complement component C8 gamma chain |
| P39061 | COIA1 Collagen alpha-1(XVIII) chain |
| P33622 | APOC3 Apolipoprotein C-III |
| P34928 | APOC1 Apolipoprotein C-I |
| O70362 | PHLD Phosphatidylinositol-glycan-specific phospholipase D |
| Q9Z2L6 | MINP1 Multiple inositol polyphosphate phosphatase 1 |
| P12399 | CTL2A Protein CTLA-2-alpha |
| Q05020 | APOC2 Apolipoprotein C-II |
| P39039 | MBL1 Mannose-binding protein A |
| P70389 | ALS Insulin-like growth factor-binding protein complex acid labile subunit |
| P25444 | RS2 40S ribosomal protein S2 |
| P41317 | MBL2 Mannose-binding protein C |
| Q9Z1R3 | APOM Apolipoprotein M |
| P63242;Q8BGY2 | IF5A1 Eukaryotic translation initiation factor 5A-1 |
| Q61268 | APOC4 Apolipoprotein C-IV |
| P09813 | APOA2 Apolipoprotein A-II |
| P07759 | SPA3K Serine protease inhibitor A3K |
| Q06770 | CBG Corticosteroid-binding globulin |
| P31532 | SAA4 Serum amyloid A-4 protein |
| P07309 | TTHY Transthyretin |

**Supplementary Table 5. Serum proteins only appeared in KO mice**

| O09173 | HGD Homogentisate 1,2-dioxygenase |
| --- | --- |
| O35664 | INAR2 Interferon alpha/beta receptor 2 |
| P00329 | ADH1 Alcohol dehydrogenase 1 |
| P00683 | RNAS1 Ribonuclease pancreatic |
| P02463 | CO4A1 Collagen alpha-1(IV) chain |
| P05201 | AATC Aspartate aminotransferase, |
| P07091 | S10A4 Protein S100-A4 |
| P09055 | ITB1 Integrin beta-1 |
| P10493 | NID1 Nidogen-1 |
| P10518 | HEM2 Delta-aminolevulinic acid dehydratase |
| P10649;Q80W21;  P15626;P48774 | GSTM1 Glutathione S-transferase Mu 1 |
| P10810 | CD14 Monocyte differentiation antigen CD14 |
| P11352 | GPX1 Glutathione peroxidase 1 |
| P14430 | HA18 H-2 class I histocompatibility antigen, Q8 alpha chain |
| P18242 | CATD Cathepsin D |
| P22777 | PAI1 Plasminogen activator inhibitor 1 |
| P26443 | DHE3 Glutamate dehydrogenase 1, |
| P27046 | MA2A1 Alpha-mannosidase 2 |
| P31240 | PDGFB Platelet-derived growth factor subunit B |
| P49429 | HPPD 4-hydroxyphenylpyruvate dioxygenase |
| P50228 | CXCL5 C-X-C motif chemokine 5 |
| P50431 | GLYC Serine hydroxymethyltransferase, cytosolic |
| P51174 | ACADL Long-chain specific acyl-CoA dehydrogenase |
| P56395 | CYB5 Cytochrome b5 |
| P63260 | ACTG Actin, cytoplasmic 2 |
| P70428 | EXT2 Exostosin-2 |
| Q09199 | B4GN2 Beta-1,4 N-acetylgalactosaminyltransferase 2 |
| Q61425 | HCDH Hydroxyacyl-coenzyme A dehydrogenase, mitochondrial |
| Q61810 | LTBP3 Latent-transforming growth factor beta-binding protein 3 |
| Q61830 | MRC1 Macrophage mannose receptor 1 |
| Q9QUI0;Q62159 | RHOA Transforming protein RhoA |
| Q64442 | DHSO Sorbitol dehydrogenase |
| Q7TSK7 | ATL2 ADAMTS-like protein 2 |
| Q8BWT1 | THIM 3-ketoacyl-CoA thiolase |
| Q8C165 | P20D1 N-fatty-acyl-amino acid synthase/hydrolase PM20D1 |
| Q8C196 | CPSM Carbamoyl-phosphate synthase |
| Q8CAY6 | THIC Acetyl-CoA acetyltransferase |
| Q8CG19 | LTBP1 Latent-transforming growth factor beta-binding protein 1 |
| Q8VC12 | HUTU Urocanate hydratase |
| Q8VCT4 | CES1D Carboxylesterase 1D |
| Q91V64 | ISOC1 Isochorismatase domain-containing protein 1 |
| Q91YI0 | ARLY Argininosuccinate lyase |
| Q91ZX7 | LRP1 Prolow-density lipoprotein receptor-related protein 1 |
| Q9D2I5 | ARMC9 LisH domain-containing protein |
| Q9D8N0 | EF1G Elongation factor 1-gamma |
| Q9DBT9 | M2GD Dimethylglycine dehydrogenase, |
| Q9DC11 | PXDC2 Plexin domain-containing protein 2 |
| Q9EQF5 | DPYS Dihydropyrimidinase |
| Q9ES89 | EXTL2 Exostosin-like 2 |
| Q9ET01;Q9WUB3 | PYGL Glycogen phosphorylase, |
| Q9JLT4 | TRXR2 Thioredoxin reductase 2 |
| Q9QWK4 | CD5L CD5 antigen-like |
| Q9QXF8 | GNMT Glycine N-methyltransferase |
| Q9R0G6 | COMP Cartilage oligomeric matrix protein |
| Q9Z0L8 | GGH Gamma-glutamyl hydrolase |
| Q9Z2W0 | DNPEP Aspartyl aminopeptidase |
| W8DXL4 | LRIT3 Leucine-rich repeat, immunoglobulin-like domain and transmembrane domain-containing protein 3 |

**Supplementary Table 6. Serum proteins only appeared in con mice**

| P08730-1;P08730 | K1C13 Keratin, type I cytoskeletal 13 |
| --- | --- |
| O89098 | CYTF Cystatin-F |
| P01878 | IGHA Ig alpha chain C region |
| P0DP28;P0DP27 | CALM3 Calmodulin-3 |
| P11103 | PARP1 Poly [ADP-ribose] polymerase 1 |
| P34884 | MIF Macrophage migration inhibitory factor |
| P56959 | FUS RNA-binding protein FUS |
| P62751 | RL23A 60S ribosomal protein L23a |
| P67778 | PHB Prohibitin |
| Q04447 | KCRB Creatine kinase B-type |
| Q5M6W3 | CLHC1 Clathrin heavy chain linker domain-containing protein 1 |
| Q64524;Q9D2U9 | H2B2E Histone H2B type 2-E |
| Q7TPX8 | OOG2 Oogenesin-2 |
| Q8VCE1 | DJC28 DnaJ homolog subfamily C member 28 |
| P70402 | MYBPH Myosin-binding protein H |
| Q61733 | RT31 28S ribosomal protein S31 |

**Supplementary Table 7. All differential metabolites in KO mice liver (down-regulated vs. con).**

| **Description** | **Fold change** | **P value** |
| --- | --- | --- |
| L-Proline | 0.11 | 5.63E-03 |
| 5'-Deoxyadenosine | 0.12 | 8.97E-03 |
| Glycitein | 0.17 | 6.17E-03 |
| Anthranilic acid (Vitamin L1) | 0.20 | 5.31E-10 |
| beta-Homoproline | 0.20 | 7.78E-03 |
| Biliverdin | 0.22 | 1.72E-03 |
| Maltopentaose | 0.29 | 1.70E-05 |
| 3-Aminobutanoic acid | 0.29 | 9.10E-04 |
| cis-(6,9,12)-Linolenic acid | 0.32 | 1.85E-04 |
| Pyridoxine | 0.32 | 4.67E-04 |
| 7,8-Dihydrobiopterin | 0.34 | 1.30E-06 |
| N-Acetylmannosamine | 0.34 | 6.92E-05 |
| N-Acetyl-D-glucosamine | 0.35 | 2.28E-06 |
| Glycerophosphocholine | 0.35 | 3.15E-04 |
| Dephosphocoenzyme A (Dephospho-CoA) | 0.36 | 1.61E-06 |
| gamma-L-Glutamyl-L-valine | 0.36 | 3.37E-04 |
| Adenosine | 0.37 | 8.83E-08 |
| Maltotriose | 0.37 | 1.09E-05 |
| Ile-Glu | 0.37 | 3.72E-05 |
| L-Leucine | 0.37 | 4.98E-03 |
| Allopurinol riboside | 0.40 | 5.57E-09 |
| Stachyose | 0.41 | 2.74E-05 |
| Hypoxanthine | 0.42 | 1.03E-08 |
| Choline | 0.42 | 1.78E-04 |
| Cellobiose | 0.43 | 1.35E-05 |
| Adenosine monophosphate (AMP) | 0.45 | 1.18E-06 |
| Ergothioneine | 0.47 | 1.61E-07 |
| Inosine | 0.48 | 3.74E-09 |
| Adenine | 0.48 | 2.44E-06 |

**Supplementary Table 8. All differential metabolites in KO mice liver (up-regulated vs. con)**

| Description | Fold change | p-value |
| --- | --- | --- |
| Acetylcarnitine | 10.68 | 1.10E-02 |
| Lys-Leu | 10.31 | 4.52E-06 |
| Prostaglandin I2 | 5.19 | 1.39E-06 |
| L-Palmitoylcamitine | 4.53 | 2.34E-04 |
| 2-Methylbutyroylcarnitine | 3.82 | 1.77E-05 |
| Adenosine 5'-triphosphate(ATP) | 3.26 | 2.36E-07 |
| O-Phosphoethanolamine | 3.20 | 2.68E-10 |
| L-Citrulline | 3.16 | 9.01E-07 |
| Sphingomyelin (d18:1/18:0) | 2.55 | 2.28E-03 |
| Eicosapentaenoic acid | 2.51 | 2.06E-06 |
| N-Acetyl-D-Glucosamine 6-Phosphate | 2.50 | 1.59E-07 |
| Taurocholate | 2.48 | 3.00E-06 |
| Cytosine | 2.47 | 1.04E-07 |
| Xanthosine | 2.39 | 7.83E-07 |
| Eicosapentaenoic Acid ethyl ester | 2.32 | 2.48E-06 |
| Sphingosine | 2.23 | 6.24E-05 |
| 20-Hydroxyarachidonic acid | 2.16 | 1.62E-05 |
| Trans-4-Hydroxy-L-proline | 2.16 | 2.81E-02 |
| Stearoylcamitine | 2.09 | 8.50E-04 |
| Cyclohexylamine | 2.07 | 1.30E-05 |
| Uracil | 2.00 | 2.55E-07 |

**Supplementary Table 9. Antibody information**

| **Name** | **Supplier** | **Cat no.** |
| --- | --- | --- |
| Kindlin-2 | Merck Millipore | MAB2617 |
| GAPDH | ZSGB-BIO | TA-08 |
| α-SMA | Absin | Abs120451 |
| F4/80 | Cell Signaling technology | 70076 |
| OPN | R&D | AF808 |
| GSTP1 | santa cruz | sc-66000 |
| Actin | ZSGB-BIO | TA-09 |
| V5 | Cell Signaling technology | 13202 |
| Flag | Cell Signaling technology | 14793 |

**Supplementary Table 10. Primer information**

| Gene Name | Forward | Reverse |
| --- | --- | --- |
| TNFα | CCACGTCGTAGCAAACCACC | GATAGCAAATCGGCTGACGG |
| MCP1 | CACTCACCTGCTGCTACTCA | GCTTGGTGACAAAAACTACAGC |
| IL-1β | TGCCACCTTTTGACAGTGATG | AAGGTCCACGGGAAAGACAC |
| IL-6 | CTCATTCTGCTCTGGAGCCC | CAACTGGATGGAAGTCTCTTGC |
| CD68 | ACTTCGGGCCATGTTTCTCTT | GGGGCTGGTAGGTTGATTGT |
| Col1a1 | TAGGCCATTGTGTATGCAGC | ACATGTTCAGCTTTGTGGACC |
| Col6a3 | ACTGGAACCACGGAAGTTCA | GTCACTTCCAACATCGAGGC |
| TGF-β1 | GTGGAAATCAACGGGATCAG | ACTTCCAACCCAGGTCCTTC |
| Timp1 | AGGTGGTCTCGTTGATTTCT | GTAAGGCCTGTAGCTGTGCC |
| Acta2 | GTTCAGTGGTGCCTCTGTCA | ACTGGGACGACATGGAAAAG |
| Fas | GCGATGAAGAGCATGGTTTAG | GGCTCAAGGGTTCCATGTT |
| Pgc1α | TCTCAGTAAGGGGCTGGTTG | TTCCGATTGGTCGCTACACC |
| TFAM | GCTCTACACGCCCCTGGTTTCTGG | TCGCTGTAGTGCCTGCTGCTCCTG |
| Keap1 | ACAACCCCATGACCAACCAG | CCGCTCTGGCTCATATCTCTC |
| Nrf2 | CGTCCCTAGGTCCTTGTTCC | ATCAAATCCATGTCCTGCTGGG |
| OPN | ATG GCT TTC ATT GGA GTT GC | GAG GAG AAG GCG CAT TAC AG |
| Kindlin-2 | TGGACGGGATAAGGATGCCA | TGACATCGAGTTTTTCCACCAAC |
| GAPDH | TTTCTTCTTGCCTTGGGAGA | AGTTCCGCACTTCATTCAGG |
| shKindlin-2-1 | CCGGGCCTTACCAGACCAGTTAATACTCG  AGTATTAACTGGTCTGGTAAGGCTTTTTG | AATTCAAAAAGCCTTACCAGACCAGTTAA  TACTCGAGTATTAACTGGTCTGGTAAGGC |
| shKindlin-2-2 | CCGGGCGGACAGTTCTTACAACTTACTCG  AGTAAGTTGTAAGAACTGTCCGCTTTTTG | AATTCAAAAAGCGGACAGTTCTTACAACT  TACTCGAGTAAGTTGTAAGAACTGTCCGC |
